# Evaluating the Utility and Impact of Canadian Plastic Surgery Residency Programs’ Instagram Accounts on Resident Recruitment and Engagement

**DOI:** 10.1177/22925503251379895

**Published:** 2025-09-29

**Authors:** Chloe R. Wong, Jacob Wise, Syena Moltaji, Heather L. Baltzer, Jeffrey Fialkov

**Affiliations:** 1 Division of Plastic, Reconstructive & Aesthetic Surgery, University of Toronto, Toronto, ON, Canada; 2 Faculty of Medicine, University of Ottawa, Ottawa, Ontario; 3 Division of Plastic Surgery, Toronto General Hospital, Toronto, ON, Canada; 4 Division of Plastic Surgery, Toronto Western Hospital, Toronto, ON, Canada; 5 Division of Plastic Surgery, Sunnybrook Health Sciences Center, Toronto, ON, Canada

**Keywords:** Plastic surgery, medical education, postgraduate medical education, internship and residency, social media, medical students, Chirurgie plastique, formation médicale, formation médicale en 3e cycle, stages et résidence, médias sociaux, étudiants en médecine

## Abstract

**Introduction:** This study assesses how Canadian Plastic Surgery Residency Instagram accounts are utilized and perceived by residents, fellows, and attending physicians, and evaluates their influence on medical students’ residency program selection. **Methods:** This 2-part study includes: (1) a descriptive analysis of Instagram activity, content, and engagement, along with a national survey of Canadian plastic surgery residents, fellows, and attendings assessing account utility; and (2) a survey of medical students who attended the University of Toronto Plastic Surgery Residency Information Session, evaluating Instagram's influence on residency selection. Descriptive statistics were reported. **Results:** Twelve of 13 Canadian Plastic Surgery Residency Programs had active Instagram accounts. Canadian Plastic Surgery Residency Instagram accounts had an average of 119 posts (SD = 94) over 5 years (SD = 2). Among surveyed residents (*N* = 27/77, 35%) and fellows/attendings (*N* = 83/328, 25%), Instagram use was reported by 93% and 81%, respectively. Resident recruitment ranked as the top goal (residents 1.75, fellows/attendings 3.17), followed by achievement highlights. Most residents (80%) and fellows/attendings (53%) felt medical students benefitted most. Preferred content included program culture (85%, 84%), resident profiles (90%, 73%), and research highlights (70%, 70%). Among medical student respondents (*N* = 25/112, 22%), 95% followed Canadian programs on Instagram, seeking program culture, resident profiles, and educational opportunities (all 89%). Over half (56%) said Instagram influenced their perception of a program, with all reporting a positive impact. **Conclusion:** Instagram is a valuable platform for Canadian Plastic Surgery Residency Programs to share insights and influence medical student decision-making.

## Résumé

**Introduction :** Cette étude évalue comment les comptes Instagram des programmes de résidence canadiens en chirurgie plastique sont utilisés et perçus par les résidents, les fellows et les médecins traitants; elle évalue leur influence sur la sélection d’un programme de résidence par les étudiants en médecine. **Méthodes :** Cette étude en deux parties comporte : (1) une analyse descriptive de l’activité sur Instagram, du contenu et de l’implication, ainsi qu’une enquête nationale sur la chirurgie plastique auprès des résidents, des fellows et des participants évaluant l’utilité des comptes, et (2) une enquête auprès des étudiants en médecine qui avaient assisté à une séance d’information sur la résidence en chirurgie plastique de l’université de Toronto, évaluant l’influence d’Instagram sur la sélection de la résidence. Des statistiques descriptives ont été produites. **Résultats :** Douze des 13 programmes canadiens de résidence en chirurgie plastique ont des comptes Instagram actifs. Les comptes Instagram de chirurgie plastique au Canada comptaient, en moyenne, 119 publications (ÉT = 94) en 5 ans (ÉT = 2). Parmi les résidents questionnés (N = 27/77, 35 %) et les fellows participants (N = 83/328, 25 %), l’utilisation d’Instagram était rapportée par, respectivement, 93 % et 81 %. Le recrutement des résidents était classé comme étant le but principal (résidents 1,75, fellows/participants 3,17), suivi par la mise en valeur des réussites. La plupart des résidents (80 %) et des fellows/participants (53 %) ont estimé que les étudiants en médecine en profitaient le plus. Le contenu préféré incluait la culture du programme (85 %, 84 %), les profils des résidents (90 %, 73 %) et les points forts de la recherche (70 %, 70 %). Parmi les étudiants en médecine ayant répondu (n = 25/112, 22 %), 95 % suivaient les programmes canadiens sur Instagram, recherchant la culture du programme, les profils de résidents et les possibilités de formation (tous 89 %). Plus de la moitié (56 %) ont déclaré qu’Instagram influençait leur point de vue sur un programme, tous indiquant un impact positif. **Conclusion** : Instagram est une plateforme utilise qui permet aux programmes canadiens de résidence en chirurgie plastique de partager des points de vue et d’influencer la prise de décision des étudiants en médecine.

## Introduction

Instagram is a ubiquitous social media platform among young adults. Its engagement levels are 30 times higher than X, formerly known as Twitter, and 67% of its users are within the ages of 18 to 29, the most common age demographic of residency training program applicants.^1,2^ Instagram is used by companies, professional organizations, journals, and residency programs alike for educational and promotional reasons.^3^ Many medical and surgical residency training programs have Instagram pages. Especially during the COVID-19 pandemic, the absence of traditional in-person activities compelled many programs to turn to social media to facilitate communication and showcase their programs.^4^ In fact, one study found that 76.9% of United States’ Ophthalmology residency training programs’ Instagram Accounts were created in 2020.^4^ Although Instagram is used by numerous residency programs for various reasons, there is a paucity in the literature assessing the utility of Canadian plastic surgery residency training program Instagram accounts.

Social media platforms may affect how prospective students choose their desired residency programs. Traditionally, medical students have sought residency programs based on factors like prestige, research opportunities, a track record of alumni success, and the prominence of faculty members.^5^ A 2017 systematic review focusing on social media in graduate medical education demonstrated that residency training programs have increasingly utilized social media platforms as a resource for educational purposes and to foster engagement among learners.^6^ In addition, these platforms have been an effective means to increase program visibility in resident recruitment.^6^ Studies have found that a large percentage of applicants in varying specialties use social media platforms to find information on residency programs, and that these platforms have a growing influence on their evaluation of the program.^7,8^ This represents a historic shift in the way that medical students make critical decisions about their residency training. While the majority of Canadian Plastic Surgery Residency programs have an active Instagram account, no study to date has investigated the impact of Canadian Plastic Surgery Instagram pages on resident recruitment.

The aims of this study were: (1) to assess the utilization and perception of Canadian Plastic Surgery Residency Instagram accounts among residents, fellows, and attending physicians and (2) to evaluate the influence of these accounts on prospective medical students’ residency program selection.

## Methods

A cross-sectional survey with 2 components was created:

Part I consists of a descriptive analysis of programmatic Instagram accounts and a survey of Canadian Plastic Surgery residents, fellows, and attending surgeons. The descriptive analysis evaluated the activity of Canadian Plastic Surgery Residency Instagram accounts, including the number of followers and accounts followed, years active, date of first post, and total number of posts. Instagram posts were classified into categories: medical, awards/publications, social events, department highlights, educational events, or miscellaneous. Follower engagement was assessed based on likes and comments.

To complement this analysis, an anonymous survey was developed by the study team to assess the use and perceptions of residency program Instagram accounts. Survey questions were informed by a review of existing literature on social media use in medical education and adapted to the context of Canadian Plastic Surgery. The survey included 3 sections: demographics, Instagram usage, and perceptions of Canadian Plastic Surgery Residency Instagram accounts (see Appendix). Content was reviewed by the study team and piloted informally with a small group of residents for clarity and relevance prior to distribution. The final survey was distributed nationally through the Canadian Society of Plastic Surgery to all resident, fellow, and attending members on October 21, 2024, and remained open for 6 weeks. Data were analyzed using descriptive statistics, with categorical variables reported as frequencies and percentages, and numerical data summarized using means and standard deviations (SD). Statistical significance was set at *P* < .05.

Part II involves an anonymous survey of medical students who attended the University of Toronto Plastic Surgery Residency Information Session. This survey also followed a structured development process, with sections covering demographics, medical student Instagram use, information gathered from Canadian Plastic Surgery Residency Instagram pages, and specific perceptions of the University of Toronto Plastic, Reconstructive, and Aesthetic Surgery Instagram account (see Appendix). It was distributed on November 6, 2024, and remained open for 6 weeks. Data were analyzed using the same statistical approach as in Part I.

This study obtained research ethics board approval (Sunnybrook Health Sciences Centre, Project 6338).

## Results

### Canadian Plastic Surgery Instagram Account Descriptive Analysis

**Figure 1.**
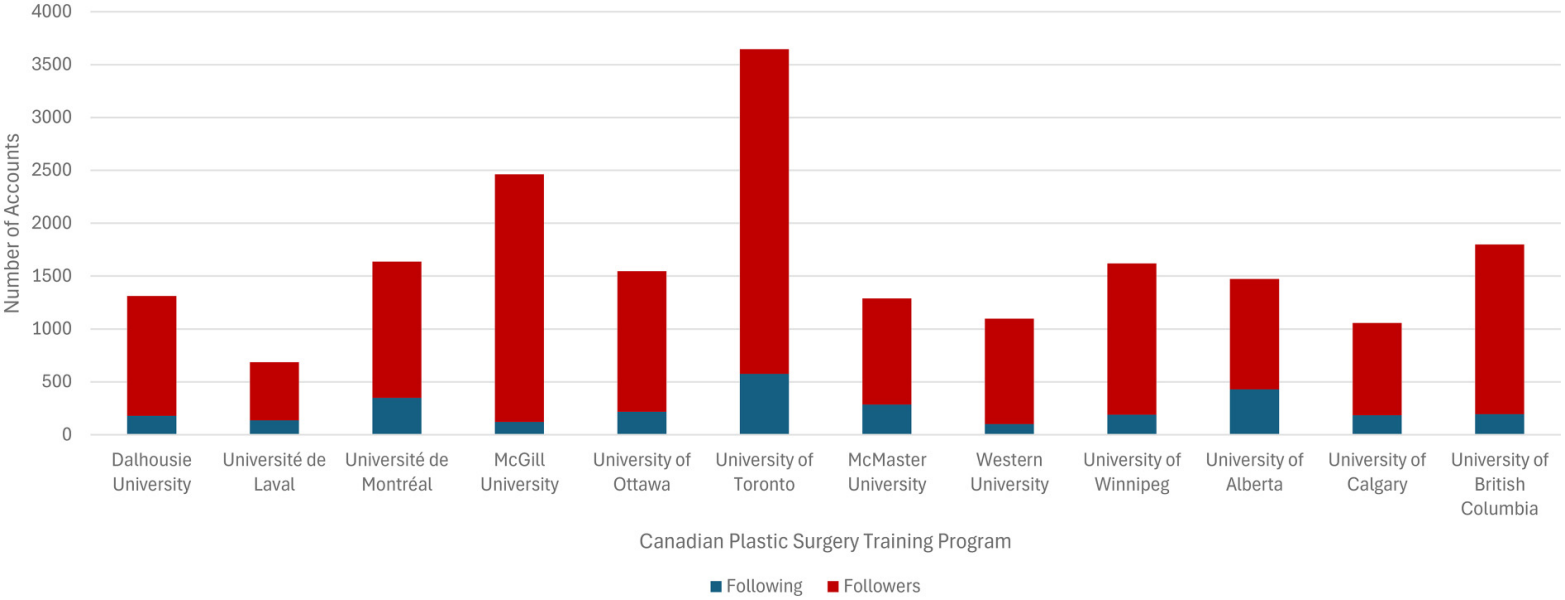
Number of accounts followed and number of followers for each Canadian plastic surgery residency training program Instagram account.

**Figure 2.**
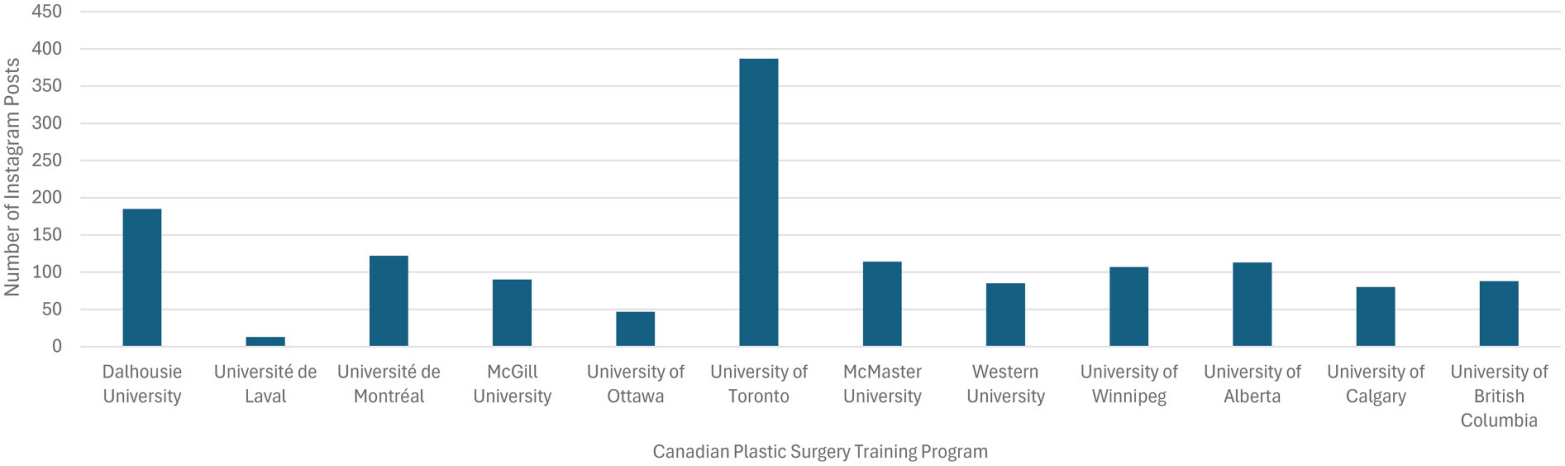
Number of Instagram posts for each Canadian plastic surgery residency training program.

**Figure 3.**
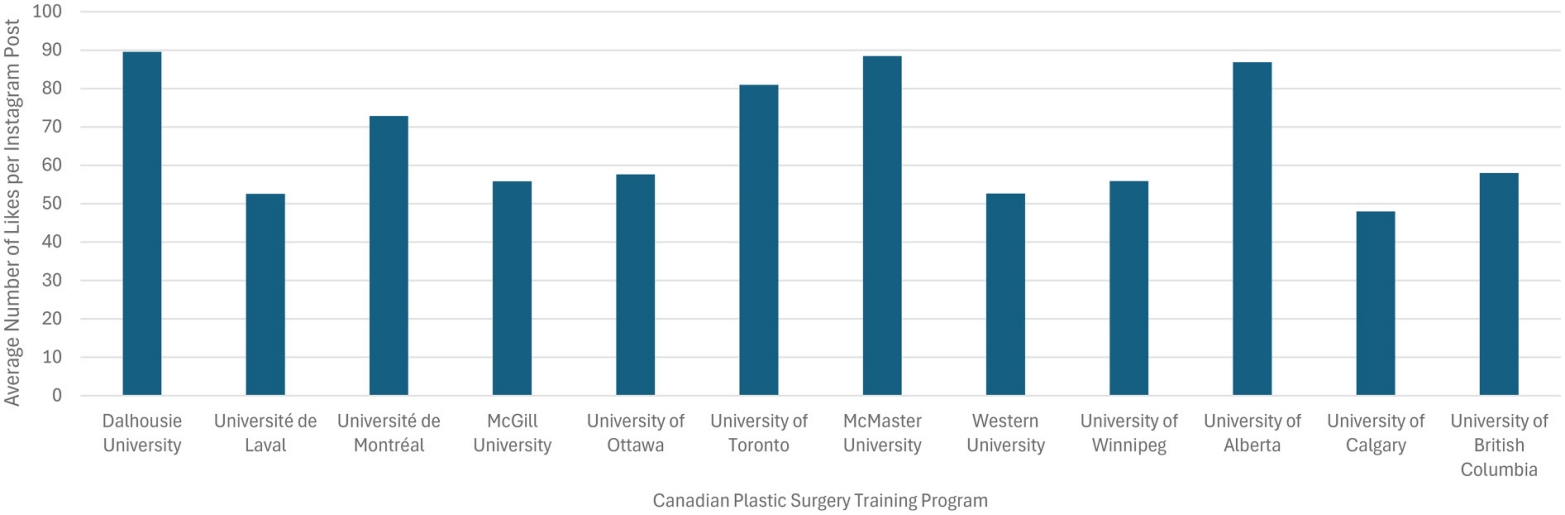
Average number of likes per Instagram post for each Canadian plastic surgery residency training program.

Of the 13 Canadian Plastic Surgery Residency Training Programs, 12 had active Instagram accounts (92%). Each account followed an average of 247 accounts (SD 141) and had an average of 1389 followers (SD 691; Figure 1). The average number of posts was 119 (SD = 94) over an average of 5 years (SD 2), with each post receiving an average of 67 likes (SD 16) and 2 comments (SD 1). See Figures 2 and 3. The most common type of post were social events, department highlights, and educational events (Table 1).

**Table 1.** Distribution of Post Categories Across Canadian Plastic Surgery Residency Instagram Accounts.

Canadian plastic surgery residency training program (number of posts)	Medical, *N* (%)	Awards/publications, *N* (%)	Social events, *N* (%)	Department highlights, *N* (%)	Educational events, *N* (%)	Miscellaneous, *N* (%)
Dalhousie University (185)	11 (6)	12 (6)	24 (13)	67 (36)	46 (25)	25 (14)
Université de Laval (12)	0 (0)	0 (0)	3 (25)	6 (50)	2 (17)	1 (8)
Université de Montréal (122)	1 (1)	10 (8)	25 (21)	39 (32)	38 (3)	9 (7)
McGill University (90)	0 (0)	10 (11)	23 (26)	27 (30)	24 (27)	6 (7)
University of Ottawa (47)	1 (2)	0 (0)	16 (34)	9 (19)	17 (36)	4 (9)
University of Toronto (386)	5 (1)	45 (12)	75 (19)	97 (25)	130 (34)	34 (9)
McMaster University (114)	0 (0)	3 (3)	42 (37)	26 (23)	33 (29)	10 (9)
Western University (91)	1 (1)	4 (4)	20 (22)	15 (17)	49 (54)	2 (2)
University of Winnipeg (107)	1 (1)	4 (4)	52 (49)	23 (22)	23 (22)	4 (4)
University of Alberta (113)	0 (0)	1 (1)	43 (38)	27 (24)	35 (31)	7 (6)
University of Calgary (61)	0 (0)	1 (2)	26 (43)	0 (0)	22 (36)	12 (20)
University of British Columbia (88)	1 (1)	3 (3)	24 (27)	26 (30)	33 (38)	1 (1)

### Assessment of the Utilization and Perception of Canadian Plastic Surgery Residency Instagram Accounts

Among surveyed residents (*N* = 27/77, 35%) and fellows/attending surgeons (*N* = 83/328, 25%), the mean age of residents was 29.5 years (SD 4.5), compared to 45.5 years (SD 11.2) for fellows and attending surgeons. Respondents included 60 males (55%), 44 females (40%), and 6 participants who identified as “other” (6%). Among those who disclosed their institution (*N* = 79/110), the distribution was as follows: University of Toronto (*N* = 32, 41%), McMaster University (*N* = 7, 9%), University of Calgary (*N* = 6, 8%), University of Manitoba (*N* = 5, 6%), Université de Montréal (*N* = 5, 6%), Dalhousie University (*N* = 5, 6%), University of British Columbia (*N* = 4, 5%), Western University (*N* = 4, 5%), Other institutions (*N* = 3, 4%), University of Alberta (*N* = 3, 4%), McGill University (*N* = 2, 3%), University of Ottawa (*N* = 1, 1%), Université Laval (*N* = 1, 1%), and Université de Sherbrooke (*N* = 1, 1%).

The distribution of postgraduate year (PGY) levels among residents was PGY-1: 15% (*N* = 5), PGY-2: 24% (*N* = 8), PGY-3: 15% (*N* = 5), PGY-4: 15% (*N* = 5), and PGY-5: 9% (*N* = 3). Among attending surgeons, 45 (60%) had an academic practice (60%) compared to 30 (40%) who had community practices. The distribution of respondents by years in practice was as follows: 0-5 years: 28% (*N* = 21), 6-10 years: 24% (*N* = 18), 11-20 years: 23% (*N* = 17), and 21+ years: 26% (*N* = 20).

Instagram use was reported by 93% of residents and 81% of fellows/attending surgeons. Among residents, Instagram is frequently used on average for 6 days a week for personal use (*N* = 21/21, 100%), followed by education (*N* = 11/21, 52%), news (*N* = 9/21, 43%), networking (*N* = 7/21, 33%), and business (*N* = 3/21, 14%). Among fellows/attending surgeons, Instagram is used on average for 6 days a week for personal use (*N* = 60/66, 91%), followed by business (*N* = 27/66, 41%), education (*N* = 26/66, 39%), news (*N* = 21/66, 32%), networking (*N* = 16/66, 24%), and other (*N* = 3/66, 5%).

Among the goals of a Canadian plastic surgery residency training program Instagram account, resident recruitment was the highest priority (weighted average on a 7-point scale: residents 1.75, fellows/attendings 3.17), followed by highlighting program achievements and awards (residents 2.35, fellows/attendings 2.69). Refer to Table 2 for further details.

**Table 2.** Goals of a Canadian Plastic Surgery Residency Training Program Instagram Account.

	Weighted average of residents (*N* = 20/27)	Weighted average of fellows/attendings (*N* = 70/77)
Resident recruitment	1.75	3.17
Patient education	6.05	5.56
Medical student/resident/fellow/attending education	3.90	4.06
Advocacy	4.80	4.49
Networking with journals, conferences, other program accounts, etc	4.75	4.39
Highlighting program achievements/awards	2.35	2.69
Showcasing research	4.40	3.66

a *Note:* weighted averages range from 1 to 7, 1 being the most important and 7 being the least important.

Most residents (*N* = 16/20, 80%) and fellows/attendings (*N* = 37/70, 53%) agreed that medical students benefitted the most from Instagram accounts (weighted average on a 4-point scale: residents 1.25, fellows/attendings 1.59). This was followed by residents (weighted average: residents 1.90, fellows/attendings 1.69). Those who benefitted the least were fellows (weighted average: residents 3.25, fellows/attendings 3.01) and attendings (weighted average: residents 3.60, fellows/attendings 3.71).

**Figure 4.**
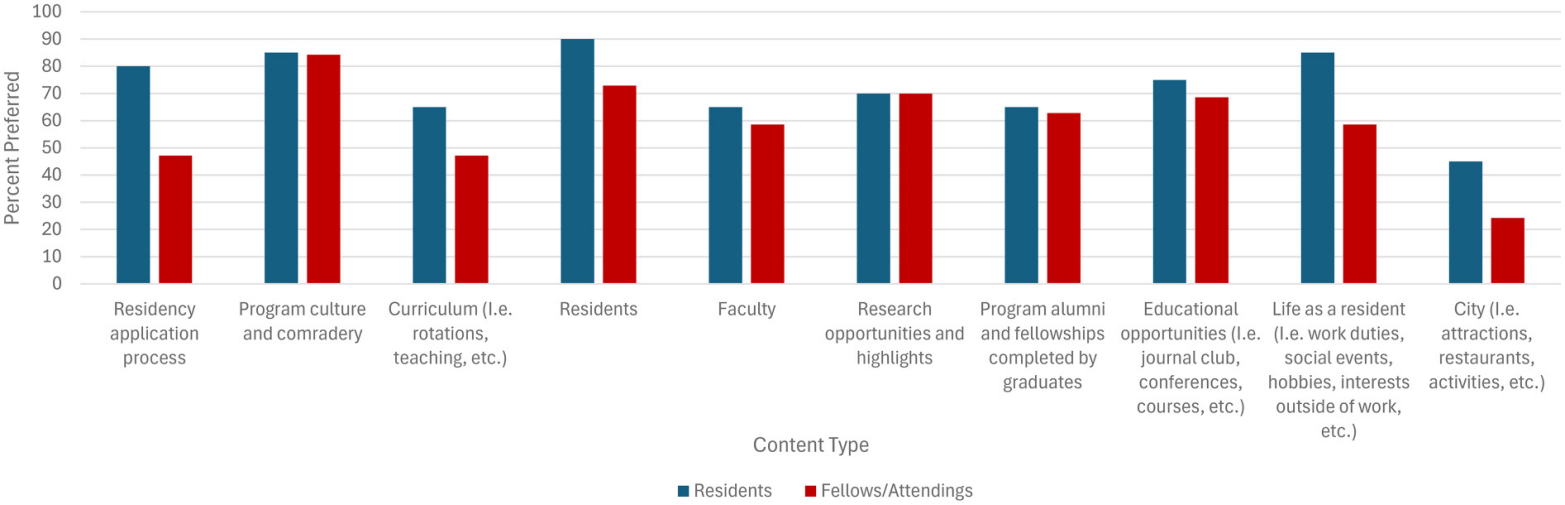
Preferred content on Canadian plastic surgery residency training program Instagram accounts, as reported by residents and fellows/attending surgeons.

Preferred content seen on Canadian plastic surgery residency training program Instagram accounts included program culture (*N* = 17/20, 85% residents; *N* = 59/70, 84% fellows/attendings), showcasing residents (*N* = 18/20, 90% residents; *N* = 51/70, 73% fellows/attendings), and research highlights/opportunities (*N* = 14/20, 70% residents; *N* = 49/70, 70% fellows/attendings; Figure 4).

In response to the question, “How can Canadian plastic surgery residency training program Instagram accounts improve?,” respondents recommended more consistent updates, inclusion of both resident and faculty perspectives, and greater emphasis on educational and research opportunities. Additional suggestions included standardizing content across programs, expanding audience engagement, and using saved stories to highlight various aspects of residency.

### Impact of Canadian Plastic Surgery Instagram Pages on Resident Recruitment

Of medical school respondents (*N* = 25/112, 22%), there was representation from the University of Toronto (*N* = 13/25, 52%), McMaster University (*N* = 5/25, 20%), University of British Columbia (*N* = 2/25, 8%), McGill University (*N* = 2/25, 8%), Western University (*N* = 1/25, 4%), University of Ottawa (*N* = 1/25, 4%), and Other (*N* = 1/25, 4%). The average age of respondents was 24.2 (SD 2.3). Respondents included 7 males (28%), 17 females (68%), and 1 participant (4%) who preferred not to disclose their gender. Respondents included: 1st-year (*N* = 7/25; 28%), 2nd-year (*N* = 7/25; 28%), 3rd-year (*N* = 4/25; 16%), 4th-year (*N* = 6/25; 24%) medical students and other (*N* = 1/25, 4%), 40% of which (*N* = 10/25) were applying to a Plastic Surgery training program in this year's Canadian Resident Matching Service cycle. Of medical student respondents, 96% (*N* = 23/24) use Instagram on an average of 6 days a week for personal use (*N* = 21/21, 100%), education (*N* = 12/21, 57%), news (*N* = 9/21, 43%), networking (*N* = 9/21, 43%), and business (*N* = 1/21, 5%).

**Figure 5.**
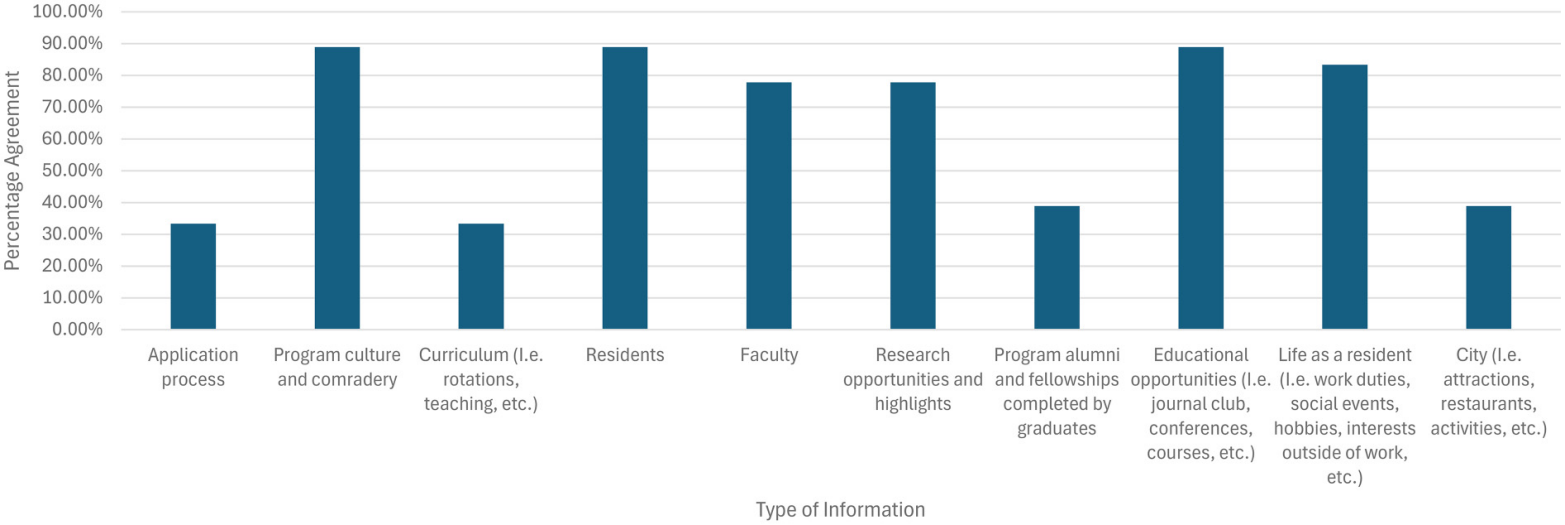
Type of information sought by medical students when browsing Canadian plastic surgery residency training program Instagram accounts.

**Figure 6.**
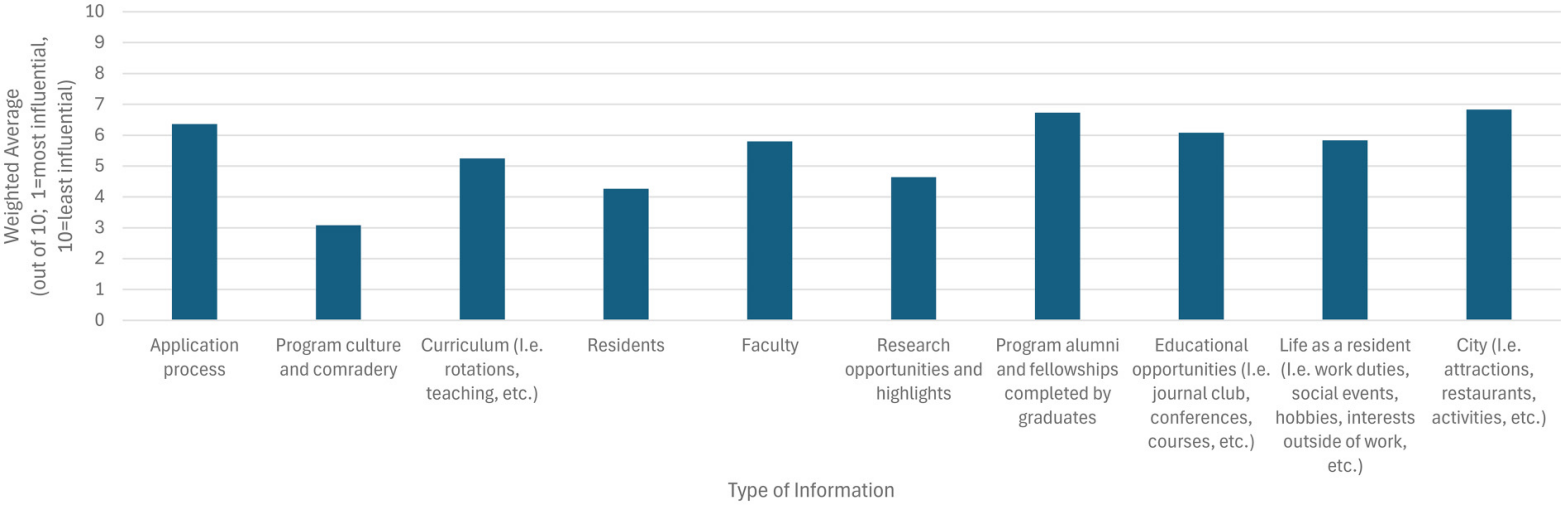
Most influential types of information on Canadian plastic surgery residency Instagram accounts, based on medical students’ perceptions of their impact on program impression. Ratings are based on a weighted average from 1 (most influential) to 10 (least influential).

When considering Canadian Plastic Surgery residency programs, 95% (*N* = 21/22) browsed or followed their respective Instagram pages to obtain information about their program. The University of Toronto Instagram page was the most frequently browsed or followed (*N* = 19/19, 100%), followed by McMaster University (*N* = 13/19, 68%), Western University (*N* = 11/19, 58%), and the University of British Columbia (*N* = 11/19, 58%). Other commonly viewed accounts included the University of Ottawa (*N* = 9/19, 47%), the University of Calgary (*N* = 8/19, 42%), and the University of Alberta (*N* = 7/19, 37%). Engagement was lower for the University of Manitoba (*N* = 4/19, 21%), McGill University (*N* = 4/19, 21%), and Dalhousie University (*N* = 4/19, 21%). The Université de Montréal and Université Laval each had the lowest engagement (*N* = 2/19, 11%). Of respondents who browsed or followed Canadian plastic surgery residency training program Instagram accounts, top information searched for included: program culture and camaraderie (*N* = 16/18, 89%), residents (*N* = 16/18, 89%), and educational opportunities (*N* = 16/18, 89%; Figure 5). When ranking the information that was most influential in terms of the perception of the program on a scale from 1 (most useful) to 10 (least useful), information about program culture and camaraderie (weighted average 3.08), residents (4.27), and research opportunities and highlights (4.64) were ranked most influential (Figure 6).

The frequency of Instagram stories or posts did not affect the perception of or decision to apply to the program (*N* = 12/19, 63%). However, over half of respondents (*N* = 10/18, 56%) indicated that the information provided on Canadian Plastic Surgery residency program Instagram pages influenced their perception of and decision to apply to the program, with an average rating of 5.84/10, 100% of which (*N* = 17/17) described a positive influence.

**Figure 7.**
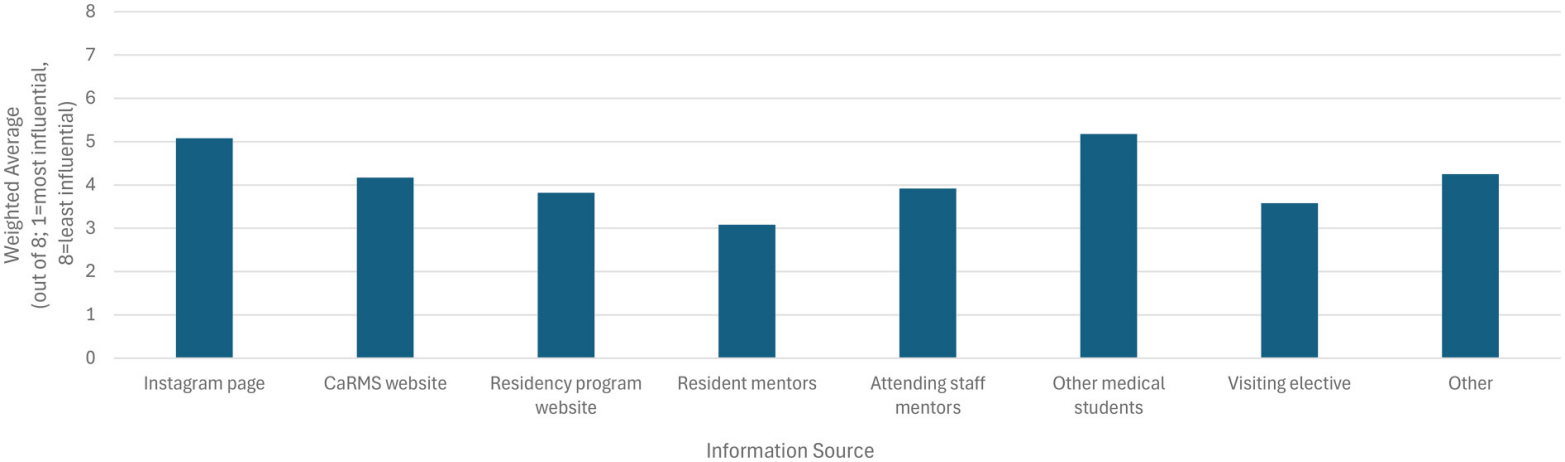
Perceived usefulness of different information sources for learning about Canadian plastic surgery residency programs. Sources were ranked from 1 (most useful) to 8 (least useful) using a weighted average.

When ranking the usefulness of different information sources on Canadian Plastic Surgery residency programs on a scale from 1 (most useful) to 8 (least useful), resident mentors were rated the most useful (weighted average: 3.08; Figure 7). The influence of Instagram pages (5.08) and other medical students (5.18) ranked lower than direct mentorship and structured program websites. While the majority of respondents (*N* = 13/19, 68%) felt that Canadian Plastic Surgery residency program Instagram pages could provide more detailed information, nearly all (*N* = 18/19, 95%) still found them to be a valuable resource, with an average rating of 7.47 out of 10. When compared to a visiting elective or formal information session, information shared by Canadian plastic surgery residency training program Instagram accounts were reported to be helpful by 40% (*N* = 4/10) and 50% (*N* = 6/12) of respondents, respectively.

Overall, all respondents (*N* = 12/12) recommend that Canadian Plastic Surgery residency programs continue to use Instagram as a platform for resident recruitment. Open-ended responses indicated a desire for more educational content, research highlights, community initiatives, networking opportunities, and video-based insights into program achievements and resident experiences.

Of respondents, 93% (*N* = 13/14) viewed the University of Toronto Instagram page. Most report that the Instagram page influenced or changed their view of the program (*N* = 10/12, 83%), in a positive way (*N* = 12/12, 100%). After viewing the University of Toronto Instagram page, respondents were either more likely to apply to the program (*N* = 6/12, 50%) or no change (*N* = 6/12, 50%). Respondents ranked content on the University of Toronto Plastic, Reconstructive, and Aesthetic Surgery Instagram page on a scale from 1 (most valuable) to 5 (least valuable). CaRMS Instagram Live Information Sessions were rated the most valuable (weighted average: 2.17), followed closely by resident profiles (2.58) and research/educational content, such as journal clubs, conferences, and courses (2.5). The “64 Questions with UofT PRS” series was ranked moderately (3.0).

## Discussion

### Summary of Findings

This cross-sectional survey study found that Instagram is widely used by Canadian Plastic Surgery residency programs, with 92% maintaining active accounts. Engagement varied, with programs averaging 247 followed accounts, 1389 followers, and 119 posts over 5 years, primarily featuring social events, department highlights, and educational content. Among surveyed residents and fellows/attending surgeons, Instagram was most beneficial for medical students and residents, with resident recruitment ranked as the highest priority. In the survey of medical students, while 56% of respondents indicated that Instagram influenced their perception and decision to apply to a program, it was ranked lower in usefulness compared to resident mentors, visiting electives, and official program websites. The University of Toronto Instagram page had the highest engagement, and respondents valued content related to program culture, residents, and research opportunities. Suggested improvements included more educational content, research highlights, faculty engagement, and video-based insights into resident experiences. Despite areas for enhancement, 95% of respondents found Instagram to be a valuable resource, and all supported its continued use for residency recruitment.

### Comparison to the Literature

The use of social media in graduate medical education has grown significantly, with residency programs establishing an online presence to enhance visibility and attract applicants.^6^ A larger percentage of applicants across multiple specialties are now using social media to evaluate programs, with platforms playing an increasing role in influencing application decisions.^7,8^ For example, in a study of anesthesia residency applicants, over 50% reported that a residency-based social media presence impacted their evaluation of programs.^7^ Additionally, 42% of applicants used both social media and internet search engines to research residency programs, highlighting the importance of a strong digital presence.^7^ Similarly, in otolaryngology, 92% of applicants accessed program social media pages, primarily on Instagram (93%) and Twitter (61%), and 32% stated that social media impacted their rank list.^9^ Program accounts were found to be the most helpful in showcasing program culture and highlighting its location.^9^ Irwin et al,^10^ surveyed applicants applying to the Harvard plastic surgery residency programs and found that 97% of applicants searched online for program information, and 20% reported that a program's social media presence influenced their perception and intended rank position, with 72% of those influenced reporting a positive impact. Applicants noted that social media helped “humanize” residents, gave insight into cultural fit, and signaled a program's engagement with technology—factors that may explain why some were positively influenced despite ranking social media as a less helpful resource overall.^10^ These intangible qualities, visible through social media, appear to play a key role in shaping applicant perceptions. Our findings were similar, supporting the growing role of social media in applicant decision-making and its positive influence on program perception.

### Areas for Improvement

Instagram serves as a highly accessible and widely used platform for medical students seeking residency program information, particularly those without direct access to residents or faculty. It provides real-time insights into program culture, resident experiences, and educational opportunities, offering a more dynamic and engaging way to showcase what programs have to offer.

Survey respondents identified a need for more structured and educational content on Instagram, including research highlights, faculty engagement, and detailed insights into residency curriculum and training opportunities. While social media provides valuable real-time insights, it remains a supplementary tool rather than a replacement for more comprehensive resources, such as program websites and visiting electives. However, Silvestre et al^11^ found that plastic surgery residency websites only provided an average of 31% of desired content items, highlighting a gap in the completeness of information available through traditional platforms. Given that Instagram is widely used by medical students, residency programs should ensure their social media presence complements and enhances their website content by providing clear, structured, and informative posts that align with applicant priorities. Incorporating faculty involvement, increasing research-related posts, and using video-based content such as interviews and “day in the life” segments can enhance engagement and provide a more comprehensive view of the program. Additionally, leveraging Instagram analytics, such as engagement metrics and post reach, can help programs refine their recruitment strategies. Ensuring accurate, transparent, and informative posts can help social media serve as a valuable complement to traditional recruitment efforts, ultimately improving outreach and attracting prospective applicants.

### Limitations and Future Directions

This study is limited by a small response rate, which may affect generalizability, but it is the first study in Canada to examine Instagram's role in Plastic Surgery residency recruitment. Self-reported data may introduce bias, and engagement was not objectively measured. Future research should consider longitudinal studies or qualitative/mixed-methods approaches to explore Instagram's long-term impact on recruitment, along with Instagram analytics to better evaluate its effectiveness.

## Conclusion

Instagram plays a key role in enhancing resident recruitment, program visibility, and engagement with medical students across Canadian Plastic Surgery residency programs. As a widely valued resource, it provides unique insights into program culture, resident experiences, and educational opportunities. Optimizing content through expanded educational resources, research highlights, and faculty involvement can further strengthen its impact on residency recruitment and outreach.

## Supplemental Material

sj-docx-1-psg-10.1177_22925503251379895 - Supplemental material for Evaluating the Utility and Impact of Canadian Plastic Surgery Residency Programs’ Instagram Accounts on Resident Recruitment and Engagement Supplemental material, sj-docx-1-psg-10.1177_22925503251379895 for Evaluating the Utility and Impact of Canadian Plastic Surgery Residency Programs’ Instagram Accounts on Resident Recruitment and Engagement by Chloe R. Wong, Jacob Wise, Syena Moltaji, Heather L. Baltzer and Jeffrey Fialkov in Plastic Surgery

**Figure:** Video 1.
