# Supplementary material for: Evaluating the Utility and Impact of Canadian Plastic Surgery Residency Programs’ Instagram Accounts on Resident Recruitment and Engagement
Source: Plast Surg (Oakv). 2025 Sep 29:22925503251379895. Online ahead of print. doi: 10.1177/22925503251379895 (PMC12479458; doi:10.1177/22925503251379895)
Supplement: sj-docx-1-psg-10.1177_22925503251379895 - Supplemental material for Evaluating the Utility and Impact of Canadian Plastic Surgery Residency Programs’ Instagram Accounts on Resident Recruitment and Engagement [file sj-docx-1-psg-10.1177_22925503251379895.docx]

**Appendix**

What is the Utility of Canadian Plastic Surgery Residency Training Program Instagram Accounts?

**Part I: Demographics**

1. Age* (Free Text)
2. Gender*
   1. Male
   2. Female
   3. Non-binary
   4. Prefer not to answer
3. Are you a resident, fellow, or attending?*
   1. Resident
   2. Fellow
   3. Attending

| If resident… | If attending… |
| --- | --- |
| 1. Level of training (PGY#)*    1. 1    2. 2    3. 3    4. 4    5. 5    6. 6+    7. Fellow 2. Institution    1. University of British Columbia    2. University of Calgary    3. University of Alberta    4. University of Manitoba    5. Western University    6. McMaster University    7. University of Toronto    8. University of Ottawa    9. McGill University    10. Université de Montréal    11. Université Laval    12. Dalhousie University    13. Other | 1. What type of practice do you have?    1. Community    2. Academic   If academic…   1. Institution    1. University of British Columbia    2. University of Calgary    3. University of Alberta    4. University of Manitoba    5. Western University    6. McMaster University    7. University of Toronto    8. University of Ottawa    9. McGill University    10. Université de Montréal    11. Université Laval    12. Dalhousie University   If community or academic…   1. Number of years in practice    1. 0-5    2. 6-10    3. 11-20    4. 21+ |

**Part II: Instagram Use**

1. Do you use Instagram?
   1. Yes
   2. No

If yes to above…

1. What are your reasons for using Instagram? (Select all that apply)
   1. Personal use
   2. Business
   3. Education
   4. News
   5. Networking
   6. Other (short answer)
2. How many days of the week are you on (i.e. browsing, posting, reading etc.) on Instagram? (sliding scale 0-7)

**Part III: Utility of Canadian Plastic Surgery Residency Training Program Instagram Accounts**

1. What do you believe to be the **most important goals** of a Canadian Plastic Surgery Residency Training Program Instagram Account? Rank from 1-7 (1=the most important, 7=the least important)
   1. Resident recruitment
   2. Patient education
   3. Medical student/resident/fellow/attending education
   4. Advocacy
   5. Networking with journals, conferences, other program accounts etc.
   6. Highlighting program achievements/awards
   7. Showcasing research
   8. Other
2. Rank the utility of Canadian Plastic Surgery Residency Training Program Instagram Accounts for the following from 1-4 (1=most beneficial, 4=least beneficial)
   1. Medical students
   2. Residents
   3. Fellows
   4. Attending surgeons
3. What content do you prefer to see on Canadian Plastic Surgery Residency Training Program Instagram Accounts? Select all that apply.
   1. Residency application process
   2. Program culture and comradery
   3. Curriculum (I.e. rotations, teaching, etc.)
   4. Residents
   5. Faculty
   6. Research opportunities and highlights
   7. Program alumni and fellowships completed by graduates
   8. Educational opportunities (I.e. journal club, conferences, courses, etc.)
   9. Life as a resident (I.e. work duties, social events, hobbies, interests outside of work, etc.)
   10. City (I.e. attractions, restaurants, activities, etc.)
4. How can Canadian Plastic Surgery Residency Training Program Instagram Accounts **improve**? (Short answer)

How do Canadian Plastic Surgery Instagram pages affect resident recruitment? A cross sectional survey.

**Part I: Demographics**

1. Medical school*

2. Age*

- 1. <24
  2. 25-29
  3. 30-34
  4. 35-39
  5. >40

3. Gender*

- 1. Male
  2. Female
  3. Non-binary
  4. Prefer not to answer

1. Level of training (MS#)*
   1. 1
   2. 2
   3. 3
   4. 4
2. Are you applying to a Plastic Surgery program in this year’s CaRMS iteration?*
   1. Yes
   2. No

**Part II: Medical Student Instagram Use**

1. Do you use Instagram?
   1. Yes
   2. No

If yes to above…

1. What are your reasons for using Instagram? Select all that apply
   1. Personal use
   2. Business
   3. Education
   4. News
   5. Networking
   6. Other (short answer)
2. How many days of the week are you on (i.e. browsing, posting, reading etc.) on Instagram? (sliding scale 0-7)

**Part III: Information from Canadian Plastic Surgery Instagram Pages**

1. When considering Canadian Plastic Surgery residency programs, did you browse/follow their Instagram page to obtain information about their program?
   1. Yes
   2. No

If no…

1. What is your reasoning for not browsing/following Canadian Plastic Surgery residency program Instagram pages? (Select all that apply)
   1. Did not know about the pages
   2. Information is not useful
   3. Did not want to follow with personal account
   4. Do not have a personal account
   5. No reason
   6. Other (short answer)
2. What information would you look for on Canadian Plastic Surgery Instagram pages? Select all that apply.
   1. Application process
   2. Program culture and comradery
   3. Curriculum (I.e. rotations, teaching, etc.)
   4. Residents
   5. Faculty
   6. Research opportunities and highlights
   7. Program alumni and fellowships completed by graduates
   8. Educational opportunities (I.e. journal club, conferences, courses, etc.)
   9. Life as a resident (I.e. work duties, social events, hobbies, interests outside of work, etc.)
   10. City (I.e. attractions, restaurants, activities, etc.)

If yes…

1. Of the Canadian Plastic Surgery Instagram pages, which have you browsed/do you follow? Select all that apply.
   1. University of British Columbia
   2. University of Calgary
   3. University of Alberta
   4. University of Manitoba
   5. Western University
   6. McMaster University
   7. University of Toronto
   8. University of Ottawa
   9. McGill University
   10. Université de Montréal
   11. Université Laval
   12. Dalhousie University
2. What information were you looking for on Canadian Plastic Surgery Instagram pages? Select all that apply.
   1. Application process
   2. Program culture and comradery
   3. Curriculum (I.e. rotations, teaching, etc.)
   4. Residents
   5. Faculty
   6. Research opportunities and highlights
   7. Program alumni and fellowships completed by graduates
   8. Educational opportunities (I.e. journal club, conferences, courses, etc.)
   9. Life as a resident (I.e. work duties, social events, hobbies, interests outside of work, etc.)
   10. City (I.e. attractions, restaurants, activities, etc.)
3. Is the information provided on Canadian Plastic Surgery residency program Instagram pages **sufficient**?
   1. Yes
   2. No
4. Does the frequency of Instagram stories or posts affect your perception of/decision to apply to the program?
   1. Yes
   2. No
5. Is the information provided on Canadian Plastic Surgery residency program Instagram pages **valuable**?
   1. Yes
   2. No
6. On a scale of 1-10 (1=not at all, 10=extremely), how **valuable** is the information provided by Canadian Plastic Surgery residency program Instagram pages?
   1. Scale 1-10
7. Does the information provided on Canadian Plastic Surgery residency program Instagram pages affect your perception of/decision to apply to the program?
   1. Yes
   2. No
8. On a scale of 1-10 (1=not at all, 10=extremely), how **influential** are Canadian Plastic Surgery residency program Instagram pages in your perception of/decision to apply to the program?
   1. Scale 1-10
9. Is the influence positive or negative?
   1. Positive
   2. Negative

If negative…

1. How was your perception of the Canadian Plastic Surgery residency program influenced negatively by the Instagram page? (Short answer)

If positive or negative…

1. When exploring Canadian Plastic Surgery Instagram pages, what content influenced your perception of the program? Please rank from #1 (most influential) to #10 (least influential).
   1. Application process
   2. Program culture and comradery
   3. Curriculum (I.e. rotations, teaching, etc.)
   4. Residents
   5. Faculty
   6. Research opportunities and highlights
   7. Program alumni and fellowships completed by graduates
   8. Educational opportunities (I.e. journal club, conferences, courses, etc.)
   9. Life as a resident (I.e. work duties, social events, hobbies, interests outside of work, etc.)
   10. City (I.e. attractions, restaurants, activities, etc.)
2. Rank the **usefulness** of the following sources of information of Canadian Plastic Surgery residency programs from 1 (most useful) to 8 (least useful):
   1. Instagram page
   2. CaRMS website
   3. Residency program website
   4. Resident mentors
   5. Attending staff mentors
   6. Other medical students
   7. Visiting elective
   8. Other (short answer)
3. How **helpful** is the information shared by Canadian Plastic Surgery Instagram pages compared to…(very unhelpful/unhelpful/neutral/helpful/very helpful)
   1. A visiting elective
   2. Formal information session
4. What additional information would be valuable for Canadian Plastic Surgery residency program Instagram pages to share? (Short answer)
5. Would you recommend that Canadian Plastic Surgery residency programs continue to use Instagram as a platform for resident recruitment?
   1. Yes
   2. No
   3. Additional info:

**Part IV: University of Toronto Plastic, Reconstructive, and Aesthetic Surgery Instagram Account**

1. Have you viewed the University of Toronto Plastic, Reconstructive, and Aesthetic Surgery Instagram page?
   1. Yes
   2. No

| If yes… | If no… |
| --- | --- |
| 1. Has the University of Toronto Plastic, Reconstructive, and Aesthetic Surgery Instagram page influenced/change your view of the program?    1. Yes    2. No 2. Was the influence/change positive or negative?    1. Positive    2. Negative 3. After viewing the University of Toronto Plastic, Reconstructive, and Aesthetic Surgery Instagram page, are you more or less likely to apply to the program?    1. More likely    2. Less likely    3. No change 4. What content is the most valuable on the University of Toronto Plastic, Reconstructive, and Aesthetic Surgery Instagram page? (Scale of 1-6, 1=most influential, 6=least influential)    1. Resident profiles    2. Research/educational content (i.e. journal club, conferences, courses, etc.)    3. CaRMS Instagram Live Information Sessions    4. 64 Questions with UofT PRS    5. Features (i.e. “Pets of UofTPRS” or “Spotify Picklist”)    6. Other 5. What content would you like to see more of? (Short answer) | 1. What are your reasons for not viewing the University of Toronto Plastic, Reconstructive, and Aesthetic Surgery Instagram page?    1. Did not know about the pages    2. Information is not useful    3. Did not want to follow with personal account    4. No reason    5. Other (short answer) |
